# A Ferroptosis-Related lncRNAs Signature Predicts Prognosis of Colon Adenocarcinoma

**DOI:** 10.3390/life13071557

**Published:** 2023-07-13

**Authors:** Ying Guo, Zehao Wang, Ye Tian, Lin Li, Jing Dong

**Affiliations:** College of Animal Science and Medicine, Shenyang Agricultural University, Shenyang 110866, China; guoying184@126.com (Y.G.); as810314452@163.com (Z.W.); 2020200160@stu.syau.edu.cn (Y.T.)

**Keywords:** colon adenocarcinoma, ferroptosis, lncRNAs, immune cell infiltration, prognosis

## Abstract

(1) Ferroptosis is a type of cellular death caused by lipid-dependent iron peroxide, which plays a major role in cancer. Long noncoding RNAs (lncRNAs) are increasingly recognized as key regulating substances in ferroptosis; (2) RNA sequencing expressions and clinical data of 519 patients with colon adenocarcinoma (COAD) were downloaded from The Cancer Genome Atlas (TCGA) database. The expression levels of lncRNAs related to ferroptosis were screened with Pearson correlation analysis. Differential genes were enriched with Gene Ontology (GO) and Kyoto Encyclopedia of Genes and Genomes (KEGG) pathways. LncRNAs related to ferroptosis were determined with univariate Cox regression and multivariate Cox regression analyses, and patients with COAD were classified into high- and low-risk subgroups according to their median risk score. The prognostic value was further examined, and the association between ferroptosis-related lncRNAs (frlncRNAs) and survival in patients with high and low risks of COAD was validated. A TCGA–COAD data set was used for receiver operating characteristic (ROC) analysis and detrended correspondence analysis (DCA) to assess prediction accuracy. Finally, a nomogram was constructed to predict survival probability; (3) We obtained a model consisting of a five-frlncRNAs signature comprising AP003555.1, AP001469.3, ITGB1-DT, AC129492.1, and AC010973.2 for determining the overall survival (OS) of patients with COAD. The survival analysis and ROC curves showed that the model had good robustness and predictive performance on the TCGA training set; (4) We found that a five-frlncRNAs signature may play a potential role in anti-COAD immunity. Risk characteristics based on frlncRNAs can accurately predict the prognosis and immunotherapy response of patients with COAD.

## 1. Introduction

Colon adenocarcinoma (COAD) is the third most common malignant tumor worldwide [1,2]. The incidence of COAD has increased with the industrialization and urbanization of countries [3]. Colorectal cancer seriously affects and threatens the health of people around the world. COAD is considered the most complex cancer [4]. Currently, the treatment of COAD mainly uses surgery, radiotherapy, and chemotherapy after diagnosis. Chemotherapy mainly uses platinum drugs [5]. However, with the widespread use of platinum drugs, COAD cells gradually become resistant to cisplatin drugs, resulting in a poor prognosis, and this is one of the most common reasons for chemotherapy failure. Most patients are in the middle or advanced stages of cancer at initial diagnosis. Although better cancer treatment options are constantly being explored, the prognosis of patients treated with existing treatments remains poor, and the treatment is extremely painful. Thus, it is urgent to explore long noncoding RNAs (lncRNAs) related to ferroptosis for improving survival and for determining the prognoses of patients with COAD.

Iron is a crucial metal component for almost all eukaryotic cells in the living body. Iron participates in many redox reactions, including electron transfer and synthesis reactions, and is an important cofactor of various enzymes [6]. Iron possesses unpaired electrons and can accept or contribute electrons, thus exhibiting flexible coordination and redox activity [7]. Under physiological conditions through multistep regulation of cells, free and unstable iron is transformed into a stable valence state and then collected, stored, or discharged, helping the body to recycle iron [8]. Iron is involved in the regulation of many biological processes in organisms. Compared with normal cells, the growth of cancer cells is more dependent on iron, and cancer cells are more prone to iron depletion. When the iron concentration is extremely high, it can lead to peroxidation of cell membrane lipids and cell death. This is known as iron ferroptosis. Ferroptosis is a new format of regulatory cell death caused by iron-dependent lipid peroxidation [9,10]. Ferroptosis is strongly associated with the tumorigenesis and progression of cancers, and iron death by activating cancer cells is newer than other methods of cancer treatment and can be especially effective against those cancer cells that have become resistant to chemotherapy.

At the same time, lncRNAs are nonprotein-coding transcripts of more than 200 nucleotides that are distinguished from small noncoding RNAs [11]. They are widely present in various eukaryotes. Importantly, in many cases, lncRNAs have been shown to be major regulators of gene expression, and they can, therefore, play a vital role in cancer. With the progression of research, especially the implementation of the Encyclopedia of DNA Elements (ENCODE) project on the human genome in 2012, it was gradually revealed that lncRNAs are not just nonsense sequences but are closely related to tumors [12]. In addition, lncRNAs have an effect on overall survival (OS), and lncRNAs are reported to be used as diagnostic and prognostic biomarkers for OS [13]. The abnormal expression of lncRNAs can cause the occurrence and development of cancer. Further studies have shown that lncRNAs play an important role in the proliferation, differentiation, invasion, metastasis, and epithelial–mesenchymal transformation (EMT) of colorectal cancer cells. The study of ferroptosis-related lncRNAs (frlncRNAs) contributed to understanding the incidence and upgrowth of lncRNAs in COAD and predicting the OS prognosis of patients with COAD.

However, studies on the relationship between ferroptosis and COAD are limited. Therefore, the discovery of new biomarkers related to ferroptosis can not only elucidate the concrete mechanism of ferroptosis in COAD but can also have important significance for predicting the prognosis of patients with COAD. The purpose of this research was to construct a novel LncRNA associated with ferroptosis for predicting the prognosis of COAD. The relationship between these lncRNAs and clinicopathological features, immune cell infiltration, and m6A modification was also determined to provide a new target and theoretical basis for the prognosis and immune environment of patients with COAD.

## 2. Materials and Methods

### 2.1. Patient Data Acquisition

The RNA sequencing (RNA-seq) data and corresponding clinical data of 519 patients with COAD were downloaded from The Cancer Genome Atlas (TCGA) database (portal.gdc.cancer.gov). The clinical data of all patients with COAD were downloaded from TCGA. Only patients with COAD who had a clear duration and status of survival were included in the study to improve the precision of the study. Forty-one patients were excluded; therefore, data from 478 patients with COAD were available for the next stage of analysis. TCGA data are available to the public, and our research follows the TCGA data publication guidelines and access policies.

### 2.2. Certification of frlncRNAs in COAD

We identified 288 ferroptosis-related genes from the ferroptosis database accessed on 15 July 2021 (FerrDb; http://www.zhounan.org/ferrdb/operations/download.html), which contained the most detail on ferroptosis-related genes The expression of ferroptosis-related genes and corresponding lncRNAs was determined by calculating the Pearson correlation coefficient. The frlncRNAs were identified according to the standard that the *p* value was less than 0.001 (*p* < 0.001), and the absolute value of the Pearson correlation coefficient was more than 0.3 (|R| > 0.3). Gene Ontology (GO) was then used to assess the main biological characteristics related to the frlncRNAs, including biological processes (BPs), molecular functions (MFs), and cellular components (CCs).

### 2.3. Construction of a Prognostic frlncRNAs Signature

First, according to the clinical data of COAD cases in TCGA, the frlncRNAs related to overall survival were determined with univariate regression analysis. The frlncRNAs with *p* < 0.01 were analyzed using multivariate regression analysis to determine whether they could be used as independent prognostic factors. The risk score for each patient was computed according to the following formula:

Risk score=Expression(FRlncRNAn)×Coefficient(FRlncRNAn)

The patients with COAD were split into low-risk and high-risk groups in view of their median risk score.

### 2.4. Assessing the Prognostic Features of Five frlncRNAs

Univariate and multivariate Cox regression analyses assessed whether the risk scores were independent of other frequently used clinicopathological parameters, such as age, sex, TNM stage, T stage, and N stage. The R package “RMS” was then used to create a line chart. All independent prognostic factors identified with multivariate Cox regression analysis were used to construct prognostic programs to determine the probability of COAD having 1-, 3-, and 5-year OS, and the Kaplan–Meier analysis was plotted to estimate the accuracy of the actual and predicted survival rates.

### 2.5. Gene Set Enrichment Analysis and Functional Enrichment Analysis

The R package “edgeR” was used to identify the genes that were significantly different between the high-risk and low-risk groups with a false discovery rate (FDR) < 0.05 and log2 fold change (|log2FC|) > 1. The functional enrichment analysis included the Gene Ontology (GO) and Kyoto Encyclopedia of Genes and Genomes (KEGG) pathways. The signaling pathways associated with the frlncRNAs signature were identified as those with *p* < 0.05 in the functional enrichment analysis.

### 2.6. Immune Infiltration Level Analysis and N6-Methyladenosine Analysis

Studies found that immune cells infiltrating the tissue around tumors effectively predict the prognosis of cancer patients. The TIMER, CIBERSORT, CIBERSORT-ABS, QUNANTISEQ, Microenvironment Cell Populations-counter (MCP-counter), XCELL, and Estimating the Proportion of Immune and Cancer cells (EPIC) tools were utilized to evaluate the proportion of immune cell infiltration in the two groups that depended on the frlncRNAs signature. Immune checkpoints (ICs) are important regulators of the immune system. It has been shown that the expression level of IC-related genes may be related to the IC treatment response. Thus, the R package “limma” was utilized to analyze 13 IC therapy–related targets (TNFSF14, TNFSF4, BTLA, LAIR1, TNFRSF4, HAVCR2, CD200, TNFRSF25, CD48, TNFRSF14, CD200R1, LAG3, and CD27) in COAD to research the potential role of frlncRNAs in IC therapy.

In addition, N6-methyladenosine (m6A) is an important factor regulating immune response and the tumor immune microenvironment (TIME). Therefore, we tested whether there were differences in the expression of 12 m6A-related genes (RBM15, YTHDC1, ZC3H13, YTHDF1, METTL3, ALKBH5, WTAP, METTL14, FTO, YTHDC2, YTHDF2, and HNRNPC) between the two groups for COAD in order to infer the relationship between m6A-related genes and the prognosis of patients with COAD.

## 3. Results

### 3.1. Identification of Differentially Expressed frlncRNAs in Patients with Colon Cancer

**Figure 1:**
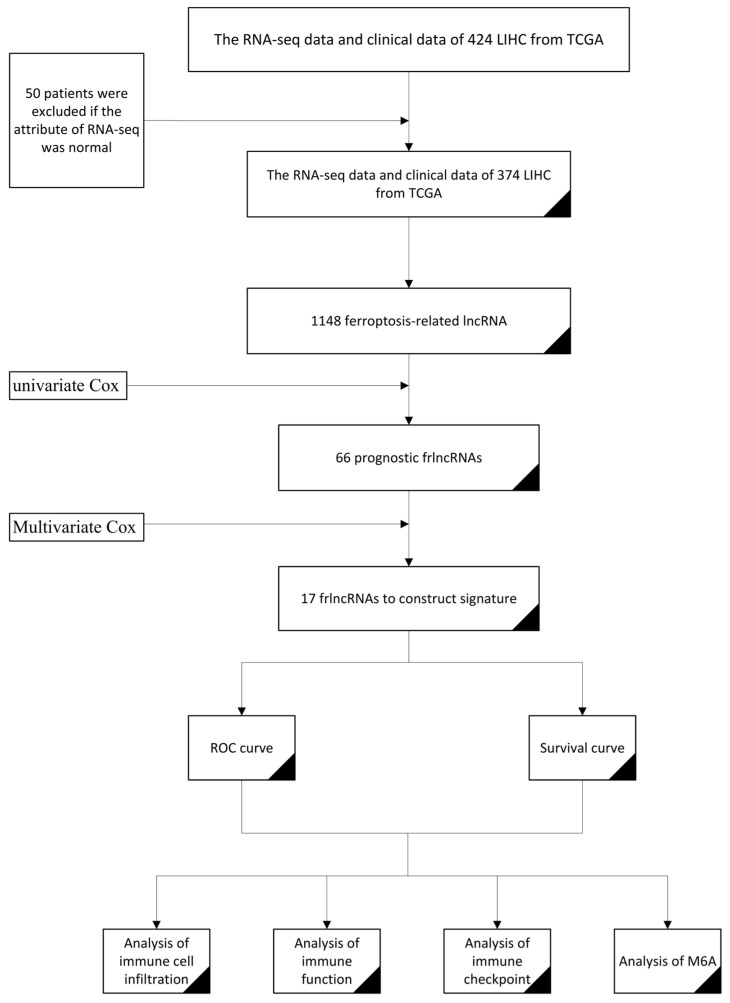
The flow chart of the construction of the frlncRNA model for predicting the prognosis of COAD.

We collected data from 519 patients with COAD from the TCGA database, which included 478 clinical patients’ data. Through difference analysis, 108 upregulated genes and 69 downregulated genes were analyzed (FDR < 0.05, log2FC > 1). Finally, we found that five lncRNAs (AP003555.1, AP001469.3, ITGB1-DT, AC129492.1, and AC010973.2) exist in differentially expressed lncRNA, prognostic lncRNA, and lncRNA related to ferritin. This means that the expression of these five genes differs between normal and tumor cells, and they determine the prognosis of patients with COAD (Figure 1).

### 3.2. Construction of a Prognostic Model

**Figure 2:**
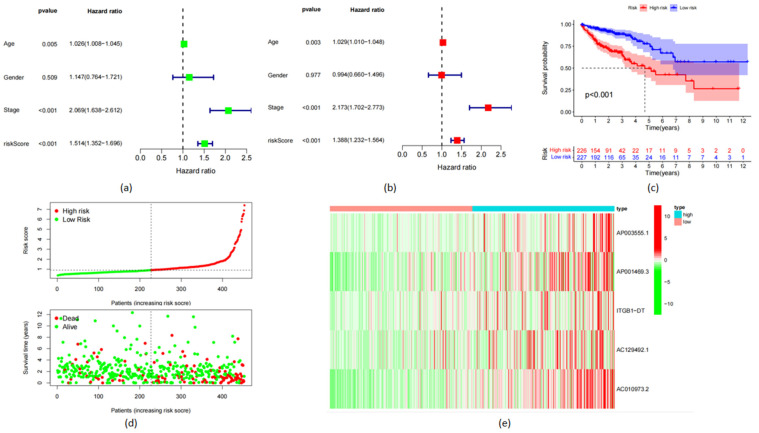
Risk Assessment and Survival Analysis. (**a**) The survival–risk score associated with age, gender, and stage; (**b**) Independent prognostic evaluation of COAD; (**c**) The survival–risk map; (**d**) The risk heatmap of the relationship between the risk index and survival rate. (**e**) frlncRNAs are positively correlated with the risk index.

The prognostic characteristics related to OS were constructed using univariate regression analysis according to frlncRNA expression indexes. Models for 17 frlncRNAs (AL161729.4, LINC00174, AP003555.1, MYG1-AS1, AC008760.1, AC011462.4, AP001469.3, AP006621.2, AC018653.3, ITGB1-DT, AC073283.1, AC129492.1, AC005841.1, AC010973.2, AC003101.2, AC008105.1, and ZKSCAN2-DT) were determined (Figure 2a). Multivariate regression analysis was performed on the results of the univariate regression analysis to obtain independent prognostic genes for COAD (Figure 2b). The following formula was used to evaluate the risk index:

Risk index (frlncRNAs signature) = (1.338 × AP003555.1) + (1.566 × AP001469.3) + (2.088 × ITGB1-DT) + (1.429 × AC129492.1) + (1.858 × AC010973.2) (1)

According to the risk score, patients in the high-risk group had higher mortality rates (Figure 2c). The Kaplan–Meier analysis showed that patients in the high-risk group had a lower OS (*p* < 0.001; Figure 2c). The survival–risk map showed a declining survival rate as the risk index increased (Figure 2d). According to the risk heat map, the expression of a five-frlncRNAs signature was positively correlated with the risk level (Figure 2e).

### 3.3. Important Pathways Identified with Enrichment Analysis

**Figure 3:**
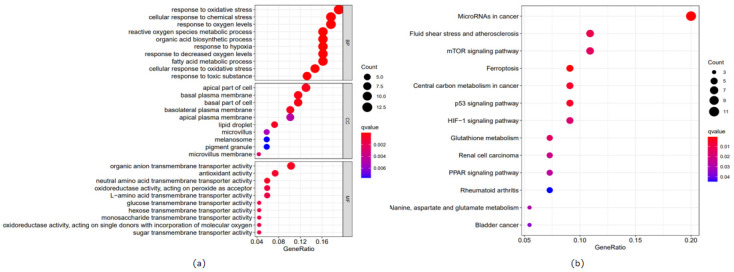
GO and KEGG analysis results. (**a**) GO analysis results. BP: immune-related biological processes. CC: immune-related cellular components. MF: immune-related molecular functions. The dot color represents the q-value. The more red, the smaller the q-value. The size of the dots represents the number of genes. The larger the number, the higher the number of genes. (**b**) KEGG analysis revealed that differentially expressed genes were enriched in immune-related pathways.

The enrichment of GO indicated that these differentially expressed ferroptosis-related genes are involved in many immune-related biological processes, including the cellular response to oxidative stress, chemical stress, and oxygen levels (Figure 3a). The differentially expressed genes also had higher representation in many immune-related cells, including the apical part of cells, the basal plasma membrane, and the basal part of cells (Figure 3a). In terms of molecular functions, the differently expressed genes were more abundant in several immune-related molecular functions, such as organic anion transmembrane transport activity, antioxidant activity, and neutral amino acid transmembrane transporter activity (Figure 3a). The KEGG pathway analysis results also showed that the differentially expressed genes were significantly enriched in immune-related pathways, including microRNAs in cancer, fluid shear stress and atherosclerosis, and the mTOR signaling pathway (Figure 3b).

### 3.4. Verification of the Accuracy of the frlncRNAs Model

**Figure 4:**
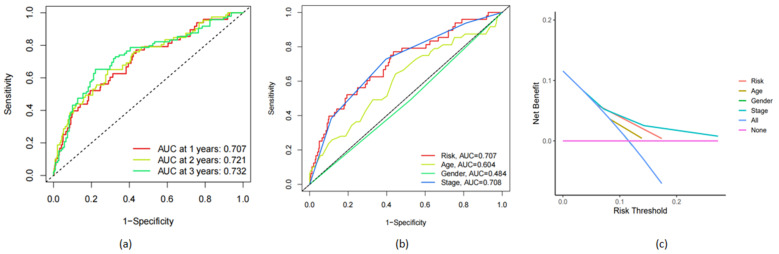
Accuracy of prognostic models. (**a**) The area under the ROC curve (AUC) validated the prognostic accuracy of the risk score. (**b**) AUC of the ROC curve indicates the prognostic accuracy of the risk score and other prognostic factors. (**c**) The decision curve analysis (DCA) shows the accuracy of the model.

The area under the ROC curve (AUC) values reached 0.707 in the first year, 0.721 in the second year, and 0.732 in the third year (Figure 4a). The AUC values used to judge the prognosis based on the traditional clinical data of risk, age, gender, and tumor stage were 0.707, 0.604, 0.484, and 0.708, respectively (Figure 4b). This shows that our model is more accurate and reliable than other clinical data for judging prognosis. Each clinical trait can be given a score, which can be summed to obtain a comprehensive score to predict the probability of survival. Similarly, we used decision curve analysis (DCA) to evaluate the prognostic value of the five-frlncRNAs characteristics and also showed that our risk model was superior to those that use other factors. This also shows that the risk index model we developed is more accurate than the traditional prognosis model (Figure 4c).

### 3.5. Immunoassay and m6A Analysis

**Figure 5:**
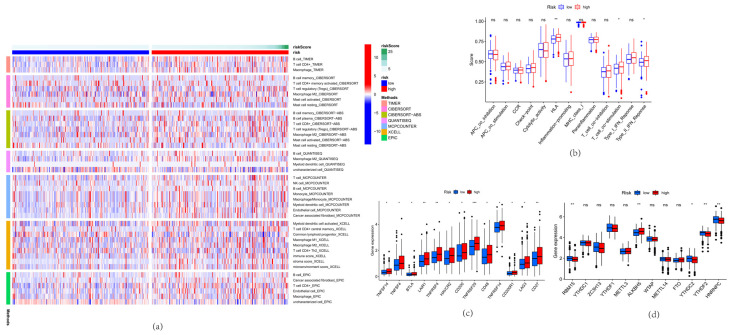
Immunoassay and m6A analysis. (**a**) Immune cell infiltration in the low-risk and high-risk groups; (**b**) Differences in immune function between the low-risk and high-risk groups; (**c**) The IC therapy-related targets; (**d**) The m6A-related genes. * indicates a significant difference (*p* < 0.05), ** indicates a highly significant difference (*p* < 0.01), *** indicates extremely significant difference (*p* < 0.001) and ns indicates no significant difference.

We used the TIMER, CIBERSORT, CIBERSORT-ABS, QUANTISEQ, MCP-counter, XCELL, and EPIC algorithms to calculate immune cell infiltration in the patients in the low-risk and high-risk groups (Figure 5a). The immune heat map showed that, compared with patients in the low-risk group, the expression of B cells, CD^8+^ cytotoxic T lymphocytes (CTLs), macrophages, and regulatory T cells (Tregs) in the high-risk group increased, and the expression of T helper cells decreased. In terms of overall immune level and tumor microenvironment (TME), the low-risk group was more immunogenic. In terms of immune function, we found significant differences between the two groups (Figure 5b). The high-risk group had higher immune function than the low-risk group for APC_co_stimulation, Check-point, HLA, Parainflammation, T_cell_co-inhibition, T_cell_co-stimulation, Type_I_IFN_Reponse, and Type_II_IFN_Reponse. We analyzed the frlncRNAs and 13 IC therapy–related targets (TNFSF14, TNFSF4, BTLA, LAIR1, TNFRSF4, HAVCR2, CD200, TNFRSF25, CD48, TNFRSF14, CD200R1, LAG3, and CD27; Figure 5c). The expression levels of TNFSF14, TNFSF4, BTLA, LAIR1, TNFRSF4, and HAVCR2 were higher in the high-risk group than in the low-risk group (Figure 5c). We screened the risk models of 12 m6A-related genes (RBM15, YTHDC1, ZC3H13, YTHDF1, METTL3, ALKBH5, WTAP, METTL14, FTO, YTHDC2, YTHDF2, and HNRNPC) to predict the OS in patients with COAD. The results showed that the expression of m6A-related genes in the low-risk group was lower than that in the high-risk group (*p* < 0.001). The expression levels of ALKBH5, METTL3, and FTO in the high-risk group were higher than those in the low-risk group. The expression levels of RBM15, YTHDC1, ZC3H13, YTHDF1, WTAP, METTL14, YTHDC2, YTHDF2, and HNRNPC were higher in the low-risk group than in the high-risk group (Figure 5d).

## 4. Discussion

The high incidence rate of COAD is related to genetic and nongenetic (environmental and lifestyle) factors. Fifty percent of patients will go on to have distant metastasis after treatments known as metastatic colorectal cancer (mCRC) with a high mortality rate [14]. Even in recent years with continuously improved treatment schemes and the development of new treatment methods, there are few effective treatment strategies due to the heterogeneity of tumors, and the prognosis of patients with advanced COAD is adverse [15]. Therefore, it is necessary to develop new and effective methods to treat COAD so as to reduce the mortality of COAD and improve the prognosis of patients with COAD. The induction of ferroptosis in tumor cells is a relatively well-established emerging cancer therapy.

In this study, we aimed to analyze COAD-associated lncRNA sequences obtained with high-throughput sequencing technology and identify frlncRNAs associated with COAD prognosis using univariate and multivariate risk regression analyses. We found that the prognostic model constructed using the AP003555.1, AP001469.3, ITGB1-DT, AC129492.1, and AC010973.2 genes was remarkably connected to the prognosis of patients with COAD. The model can independently predict the prognosis of patients with COAD. The increase in these five frlncRNAs in the high-risk group proves that their expression is unfavorable for the prognosis of patients with COAD, and we can reasonably speculate that the increase in these five frlncRNAs will promote the development of cancer cells. At present, studies show that ITGB1-DT is an lncRNA associated with a poor prognosis in COAD, and the increased expression of ITGB1-DT in COAD tissues and cells promotes the proliferation and metastasis of COAD cells [16]. In non-small cell lung cancer (NSCLC), inhibition of ITGB1-DT expression inhibits cancer development and enhances cisplatin sensitivity in NSCLC via the MAPK/ERK pathway. Inhibiting the expression of ITGB1-DT in gastric adenocarcinoma can help to inhibit the development and metastasis of gastric adenocarcinoma and reduce the occurrence of poor prognosis [16]. In lung adenocarcinoma, ITGB1-DT can also serve as a diagnostic marker and can regulate the ARNTL2 and Wnt/β-catenin pathways to influence the immune microenvironment and the development of lung adenocarcinoma [17]. AP003555.1 has not been studied in other cancers, and knocking down AP003555.1 inhibits ferroptosis in COAD [18]. AC129492.1 is associated with prognosis in osteosarcoma-related studies. AC010973.2 is considered to be one of the dry-related genes that can regulate the development of renal clear cell carcinoma. AP001469.3 was found to be associated with poor survival in patients with hepatocellular carcinoma. In summary, our prognostic model seems to be feasible. These frlncRNAs are very important for the occurrence and development of COAD, and targeted therapy may greatly improve the prognosis of patients with COAD.

According to the prognostic model, the patients were divided into high-risk and low-risk groups according to their risk scores. Differentially expressed genes between the high-risk and low-risk groups were analyzed. According to the gene functional enrichment analysis (GO and KEGG), there were noteworthy differences in immune-related pathways between the two groups, including microRNAs in cancer, fluid shear stress and atherosclerosis, and the mTOR signaling pathway. MicroRNAs are important regulators in biological processes and are involved in cell development, angiogenesis, differentiation, proliferation, survival, and immune function [19]. MicroRNAs have both oncogenic and tumor-suppressor functions. MicroRNAs can be used to diagnose and predict the prognosis of cancer. Our results also showed that microRNAs were enriched in KEGG in patients with colorectal cancer. Fluid shear stress is one of the important cellular mechanical forces that can maintain the survival of colon cancer cells [20]. Due to the fluid shear stress exerted by blood flow on endothelial cells, fluid shear stress plays a vital role in the initiation and development of atherosclerosis. The serine/threonine protein kinase mTOR occurs in many human cancers [21]. The mTOR pathway is closely related to the proliferation, invasion, and apoptosis of colorectal cancer cells. Its activation occurs frequently in various human cancers and has been demonstrated to be an anticancer therapeutic target. Inhibition of the mTOR signaling pathway induces autophagy in colorectal cancer cells, whereas inhibited mTOR signaling attenuates colorectal cancer cell migration and invasion by rapamycin, an mTOR inhibitor. In addition, the mTOR pathway can induce resistance to chemotherapy drugs by regulating angiogenesis. Abnormal activation of the mTOR signaling pathway can promote the proliferation, invasion, and metastasis of colorectal cancer cells.

We performed a survival analysis and distinguished between the high-risk and low-risk groups based on the risk scores. In addition, in order to verify the accuracy of the prediction model we built, we drew the ROC curve to calculate the AUC and the decision curve. The results show that the prognostic model we constructed is more accurate in predicting the survival rate of patients with COAD than clinical staging. Univariate and multivariate Cox regression analyses proved that the risk model we constructed can be used as an independent prognostic indicator.

Tumor treatment includes four categories: surgical treatment, radiotherapy, chemotherapy, and immunotherapy (IC). At present, IC therapy has demonstrated strong antitumor activity and has achieved clinical breakthroughs in the treatment of solid tumors, such as melanoma, non-small-cell lung cancer, renal cancer, and prostate cancer [22]. Ferroptosis is closely related to tumor immunity. TME is a dynamic system that is widely distributed in various tumor-related immune cells, such as tumor-infiltrating lymphocytes (TILs), cytotoxic T lymphocytes (CTLs), regulatory T cells (Tregs), tumor-associated macrophages (TAMs), and bone marrow-derived suppressor cells (MDSCs). These cells play an important role in the occurrence and development of COAD [23]. Immune checkpoint blocking is the most well-studied class of immunotherapy that rejuvenates T cells and blocks immune escape. PD-1 inhibitors are clinically used to relieve colorectal cancer and function as drug combinations. The lymphocytes infiltrating into the tumor are called TILs, which mediate the TME of immunosuppression. TILs have achieved good results in the evaluation of tumor response in breast cancer and improved the accuracy of patient prognosis. The TILs also help tumor cells realize immune escape and promote the malignant development of tumors. A study of 21,015 patients with COAD found that extensive tumor inflammatory cell infiltration can prolong the survival of patients with colorectal cancer. Blocking the differentiation of TILs can inhibit tumor growth [24]. CTLs, as a key component of the adaptive immune system, can cause a targeted immune response after activation. CTLs can be secreted lysosomes to inhibit tumor activity and inhibit tumor development and metastasis [25]. It has been proven that the expression of CTLs can predict the prognosis of patients with colorectal cancer. High macrophage infiltration is associated with a poor prognosis in patients with COAD. Studies show that targeting TAMs can enhance the anti-PD-1 therapeutic effect of colorectal cancer [26]. The recruitment of TAMs and Tregs in colorectal cancer can signal the transducer and activator of the transcription 3 signaling pathway to induce tumor immunosuppression [27]. Inhibition of Treg cells can enhance resistance to the colorectal cancer immune response. In this regard, our results also showed that, compared with patients in the low-risk group, the expression of B cells, CTLs, and Tregs in the high-risk group was increased. ICs as key regulators are able to regulate the immune system. ICs can induce an immunosuppressive TME and a tolerance to tumor antigens. On the one hand, ICs can awaken the immune system to respond to host antigens to protect healthy tissue [28]. On the other hand, ICs can be responsible for the escape of tumor cells [29]. As a negative regulator, they are expressed in tumors and promote the expansion of cancer cells [30]. Therefore, the single use or adjuvant therapy of IC therapy has become the mainstay in the current treatment of cancer and has broader prospects. IC inhibitors can improve the antitumor immune response of patients’ immune systems. Currently, IC inhibitors combined with chemotherapy are commonly used in clinics to improve chemotherapy response, overcome immunosuppression, produce more effective antitumor effects, and improve clinical efficacy. The expression levels of TNFSF14, TNFSF4, BTLA, LAIR1, TNFRSF4, and HAVCR2 in the high-risk group were higher than those in the low-risk group. We can speculate that these six genes can promote the proliferation and metastasis of tumor cells and may inhibit the immune system. Tumor necrosis factor superfamily member 14 (TNFSF14; also known as LIGHT) acts as an enhanced cancer immunotherapeutic agent to fight cancer. TNFSF14 binding to T cell-containing bis-specific antibodies can generate anti-colorectal cancer immunity, inhibit colorectal cancer metastasis, and thus treat colorectal cancer based on IC [31]. The combination of LIGHT and interleukin-2 increased CTLs in mice with COAD achieved a long-lasting, anti-tumor ability. In addition, LIGHT plays an important role in the resolution of inflammation caused by innate immune cells. Tumor necrosis factor superfamily member 4 (TNFSF4; also known as OX40L) is a cytokine that promotes T cell activation and proliferation. Elevated levels of TNFSF4 have been found in patients with gastric cancer, esophageal cancer, liver cancer, and breast cancer, which suggests that TNFSF4 may be an effective candidate for cancer treatment. TNFSF4 can promote the chemotherapy resistance of cancer cells by inhibiting tumor cell apoptosis. In mouse models, the targeted delivery of TNFSF4 increased CD8 T cells and NK cells to provide local anti-tumor immunity [32]. T lymphocyte attenuator (BTLA) is a new checkpoint co-inhibitory receptor that can inhibit immune response. Dysregulation of BTLA has been found in chronic lymphocytic leukemia, and the inhibition of BLTA in patients with chronic lymphocytic leukemia can effectively treat chronic lymphocytic leukemia. The knockout of human BTLA was found to reduce T cell-mediated antitumor response in a mouse model of COAD [33]. Leukocyte-associated immunoglobulin-like receptor 1 (LAIR-1) is highly expressed in tumor cells. Stimulating the function of LAIR-1 to inhibit tumor growth, which causes T cells to fail, results in a decreased immune response [34]. In bone recurrence of breast cancer, osteoblasts have been found to promote tumor metastasis and growth by inhibiting LAIR-1 signaling and inhibiting NK cells. However, there has been no in-depth study of LAIR-1 in COAD [35]. TNFRSF4 enhances antitumor immune activity by activating the NF-kappa-B pathway, PI3K/PKB pathway, and NFAT pathway. Anti-TNFRSF4 in mice showed that the volume of mouse lymphatic tumors decreased significantly. Patients with COAD and a high expression of TNFRSF4 had shorter survival [36]. HAVCR2 can encode T cell immunoglobulin 3. The combination of anti-TIM-3 and anti-PD-1 can inhibit tumor growth and has entered clinical trials.

The internal epigenetic modification m6A can regulate RNA transport, localization, translation, and degradation, which can be a therapeutic target for malignant tumors because of its dynamic and reversible characteristics [37]. It is involved in various disease processes, especially in the proliferation, metastasis, invasion, and angiogenesis of tumor cells. In this study, we found that the expression levels of ALKBH5, METTL3, and FTO in the high-risk group were higher than those in the low-risk group. Therefore, it can be speculated that ALKBH5, METTL3, and FTO may accelerate the growth of colorectal cancer. ALKBH5 can promote the proliferation, migration, and invasion of glioblastoma multiforme. When ALKBH5 expression is reduced, the formation of COAD is inhibited [38]. ALKBH5 activates RAB6A and inhibits YTHDF2 during m6A methylation, thus promoting the occurrence and development of COAD [39]. ALKBH5 can significantly inhibit the growth of COAD by regulating ALKBH5. METTL3 knockdown enhances the anti-COAD ability of anti-PD-1 immunotherapy through the PI3K/AKT pathway in TAM [40]. In in vitro studies in nude mice, METTL3 knockdown was found to inhibit COAD migration and epithelial–mesenchymal transition (EMT) by inhibiting DDX27 expression [41]. FTO is highly expressed in gastric cancer and negatively correlated with patient survival [42]. FTO inhibitors in COAD can effectively inhibit the proliferation, invasion, and metastasis. In addition, FTO can enhance chemotherapy resistance in colorectal cancer.

We constructed a new prognostic model of lncRNAs associated with ferroptosis, identified differential genes and enrichment pathways, and established a lncRNAs group related to immunization and m6A. This study helps to advance targeted therapy for colorectal cancer in the clinic.

## Data Availability

The RNA sequencing were downloaded from The Cancer Genome Atlas (TCGA) database (portal.gdc.cancer.gov). The authors confirm that the data supporting the findings of this study are available within the article.
